# “I am not shy anymore”: A qualitative study of the role of an interactive mHealth intervention on sexual health knowledge, attitudes, and behaviors of South African adolescents with perinatal HIV

**DOI:** 10.1186/s12978-022-01519-2

**Published:** 2022-12-01

**Authors:** Scarlett Bergam, Thobekile Sibaya, Nompumelelo Ndlela, Mpume Kuzwayo, Messaline Fomo, Madeleine H. Goldstein, Vincent C. Marconi, Jessica E. Haberer, Moherndran Archary, Brian C. Zanoni

**Affiliations:** 1 Department of Paediatrics, Nelson Mandela School of Medicine, University of KwaZulu Natal, Durban, South Africa; 2 Departments of Medicine and Pediatric Infectious Diseases, Emory University School of Medicine, Atlanta, GA USA; 3 Department of Global Health, Emory University’s Rollins School of Public Health, Atlanta, GA USA; 4 Children’s Healthcare of Atlanta, Atlanta, GA USA; 5 Massachusetts General Hospital, Boston, MA USA; 6 Harvard Medical School, Boston, MA USA; 7 George Washington School of Medicine and Health Sciences, Washington, DC USA

**Keywords:** Adolescent health, Sex education, HIV, Global health, mHealth, Gender, Sexuality, Reproductive health

## Abstract

**Background:**

South Africa has one of the highest burdens of adolescents with perinatally-acquired HIV (APHIV) in the world. APHIV in South Africa have limited access to sexual and reproductive health (SRH) education and services specific to their HIV status. When lacking comprehensive SRH education, APHIV are prone to sexual risk behaviors that can lead to unintended pregnancy, sexually transmitted infections, and HIV transmission. The use of mHealth interventions has been shown to deliver information, foster social support, and improve decision-making skills. In this study, we evaluate how an mHealth intervention influences sexual health knowledge and behaviors in APHIV.

**Methods:**

We purposively enrolled adolescents from the intervention arm of a randomized clinical trial assessing a multi-module, moderated WhatsApp-based intervention—Interactive Transition Support for Adolescents Living with HIV (InTSHA)—within a government supported clinic in KwaMashu, an urban township of KwaZulu-Natal, South Africa. We conducted in-depth interviews based on World Health Organization guidelines for asking adolescents about SRH. We thematically analyzed data through an iterative, team-based coding approach combining deductive and inductive elements to contextualize SRH attitudes, knowledge, and behaviors before and after receiving the InTSHA intervention.

**Results:**

Of the 21 participants, 13 (61.9%) were female and the mean age was 16.6 years. Most participants reported first learning about SRH as young teenagers in school through non-targeted and negative ways, seeking clarification through peers and the internet rather than clinicians or caregivers. Participants reported that InTSHA provided a holistic perspective on relationships, gender, and sexuality specific to growing up with HIV in South Africa. They praised the ability to give and receive information from peers in a moderated setting through the mHealth intervention, building their confidence, decision-making skills, and communication with partners and caregivers throughout their everyday lives. Despite reporting some technological challenges, adolescents agreed that InTSHA was convenient, confidential, and user-friendly.

**Conclusions:**

South African APHIV receive incomplete and conflicting sexual education from peers, caregivers, teachers, and technology that can be supplemented by mHealth curricula targeted for the unique needs of APHIV. Future, scaled-up mHealth interventions can lower SRH stigma by expanding access to sexual education and peer support, supplementing adolescents’ existing SRH education.

**Supplementary Information:**

The online version contains supplementary material available at 10.1186/s12978-022-01519-2.

## Plain Language summary

South African adolescents with perinatally-acquired HIV (APHIV)—teenagers who were born with HIV—lack access to sexual and reproductive health (SRH) education specific to their HIV status. Ineffective SRH education often leads to risky behaviors (such as unprotected sex), teenage pregnancy, and the spread of HIV. Mobile health (mHealth) interventions, such as phone apps, can help improve adolescent SRH knowledge and decision-making. In this study, we evaluate how a WhatsApp-based intervention—Interactive Transition Support for Adolescents Living with HIV (InTSHA)—influences SRH attitudes and behaviors of APHIV. InTSHA involved long-term, weekly group chats of APHIV, run by trained facilitators, that included two modules discussing SRH. This study analyzes in-depth interviews we conducted with adolescents who completed InTSHA, asking them about their SRH knowledge and behaviors before and after the intervention. The interviews took place in a government-supported clinic in an urban township of KwaZulu-Natal. Interviews were recorded, transcribed, translated, coded, and analyzed. Of 21 participants, 13 were female and the average age was 16.6 years. Most participants reported first learning about SRH as young teenagers in school, through peers, or on the internet, rather than through healthcare workers or family members. Participants reported that InTSHA provided a comfortable platform to discuss relationships, gender, and sexuality as APHIV. Adolescents enjoyed giving and receiving information from peers in a moderated setting. They reported that InTSHA built their confidence, decision-making skills, and communication with partners and caregivers. South African SRH education for APHIV can be supplemented by targeted mHealth interventions.

**Supplementary Information:**

The online version contains supplementary material available at 10.1186/s12978-022-01519-2.

## Introduction

South Africa has approximately 360,000 adolescents ages 10 to 19 living with HIV, over a third of all adolescents living with HIV (ALHIV) in sub-Saharan Africa (SSA) [1–3]. KwaZulu-Natal province has the highest number of ALHIV in South Africa [3–5]. An estimated one in five adolescents living with HIV across the world was perinatally infected, and 79% of adolescents with perinatally-acquired HIV (APHIV) reside in SSA [6]. Health policy has largely overlooked social health inequities among APHIV [5], who face unique risks related to their sexual and reproductive health (SRH) [7]. A 2021 scoping review identified gaps for APHIV pertaining to knowledge of SRH and access to SRH services, high rates of risky sexual practices, and low family planning uptake [8], leading to higher rates of unintended pregnancy than their HIV-negative counterparts [9]. Preventing the population-level spread of HIV requires expanding access to SRH resources and education for youth worldwide, especially South African APHIV [10].

Youth in SSA have high rates of unprotected sex, sexually transmitted infections, and unintended pregnancy, likely arising from a confluence of limited information, inadequate services, and negative peer influences [11]. Across SSA, adolescent SRH services are inequitably distributed by region, gender, education, urban–rural residence, economic status, and HIV status [12]. In a survey of South African adolescents in grades 8 to 12, the mean age for first-time sexual intercourse was 15.2 years, and 26.6% of adolescents reported being ‘sexually active’ [13]. Only 40.3% of sexually active adolescents reported contraception use [13], which is dependent on knowledge, relationship status, age difference, accessibility of contraception, and healthcare worker attitudes [14, 15]. As a result, adolescents in KwaZulu-Natal face high rates of teenage pregnancy—from 2010–2018, 16.9% of adolescent girls overall, and 37.1% of adolescent girls who were sexually active, reported having ever been pregnant [16]. Peer interactions have an established effect on youth sexual behaviors [17]. Sexual partners are often introduced to young women through female friends, and having older friends predicts having older partners [18]. Group acceptance, often leading to peer pressure, plays a role in predicting health outcomes [19]. Further, gender inequalities in APHIV can manifest as SRH inequities. Across Africa, 15–19-year-old adolescent girls have over twice the HIV prevalence of their male counterparts (3.3% and 1.5%, respectively) [12], calling for creative interventions to address this unmet need [20].

Current literature shows major gaps in sexual health knowledge and decision-making in youth across SSA, with APHIV in even greater need of targeted education and greater access to SRH resources [12]. Adolescents in South Africa, who primarily receive their SRH education in school, have high rates of unprotected sexual activity without awareness of the consequences, or the ability to communicate with partners and caregivers about access to preventive healthcare and information [13]. While peers play an integral role in adolescent sexual education, few studies have described the nuances and impacts of specific peer relationships on adolescent SRH [17, 18]. Further, across SSA, family members have been shown to create an uncomfortable environment about sexuality due to cultural and religious norms [21]. When adolescents fail to receive positive sexuality education, they often turn to the internet, where they gain unrealistic norms about sex and sexuality [22]. ALHIV in Sub-Saharan Africa, even more so than adolescents without HIV, have unaddressed needs regarding psychosocial and familial support, self-management techniques, health services, financial support, education, and social and sexual intimacy [23].

To bridge these gaps faced by all adolescents, but especially amongst adolescents who are coming of age while living with HIV, APHIV need holistic SRH education targeted to their unique health needs, including strategies to improve communication, decision-making skills, self-efficacy, and self-determination to begin to improve SRH knowledge and behavior [24]. In the realm of their sexual health, APHIV require integrated and engaging health, educational, and social services that promote holistic sexual wellness [5, 12]. Researchers, healthcare providers, and APHIV alike have recommended the use of mobile health (mHealth) interventions, such as phone applications or text message programs, to connect adolescents with sexual health education and services, due to high smartphone coverage and technological familiarity amongst young people [13, 19]. To mitigate concerns of security, liability, and regulation of mHealth services [25], the World Health Organization has created a guide that lays out standards for the content, context, and technical features of mHealth interventions [26].

mHealth interventions have been shown to either engage adolescents in HIV prevention and care [27, 28] or provide adolescent sexual education [29–34], but usually not at the same time. Many mHealth interventions have been implemented across SSA to help young people access information about HIV [35, 36] and to help healthcare systems in providing effective comprehensive HIV care [19, 27, 37]. HIV-focused mHealth interventions such as the ILoveLife website to prevent the spread of HIV in Uganda [36] and a text-based counseling intervention to increase ART adherence in APHIV in South Africa [28] showed success in improving knowledge and behavior change but were more didactic than interactive. Additionally, a mixed-methods analysis of an mHealth intervention in adult men has shown efficacy in linkage to HIV care [38]. Separately, mHealth interventions—such as targeted text-message conversations—have been used across contexts to deliver SRH information [29–31]. Recent mHealth interventions for adolescent SRH education have included MyQuestion, which allowed young Nigerians to text anonymous SRH questions to trained counselors about menstruation, pregnancy, STIs, and relationships [29]; and In This toGether, a daily text-message based program in Uganda, which provided informational support—but not peer or social support—about safe sex [34]; and Facebook groups in which young adults can discuss SRH in an unmoderated environment, making them prone to the spread of misinformation [39]. However, few mHealth interventions have been targeted for APHIV about their sexual and reproductive health [40].

InTSHA (Interactive Transition Support for Adolescents Living with HIV) was developed to deliver evidence-based, accessible information to APHIV in the form of a moderated, WhatsApp-based social support group. The intervention was designed to improve retention in care and viral suppression during the transition to adult care and included two modules about sexual and reproductive health. This study examines adolescents’ impressions on the role InTSHA played on their SRH education as APHIV. The purpose of this study was to assess (1) how urban, South African APHIV receive SRH information, and (2) how InTSHA played a role in shaping adolescents’ SRH knowledge, attitudes, and behaviors.

## Methods

### Study setting

The study took place in a government supported HIV clinic in KwaMashu, a large urban township of eThekwini, South Africa, located in the country’s KwaZulu-Natal province. The health center at which our study took place exclusively serves people living with HIV in the KwaMashu community, with a catchment population of about 750,000. APHIV at the clinic are treated alongside adult patients and visit the clinic monthly to collect medication and attend appointments with nurses. There are no organized SRH education or peer group programs at the clinic.

### InTSHA parent study

Between March 2021 and February 2022, we recruited 80 adolescents to the InTSHA trial who were 15–19 years old; perinatally infected with HIV; receiving ART for at least 6 months; fully aware of their HIV status; and able to access a smartphone. We excluded adolescents who were unable to read and/or speak English or *isi*Zulu or unable to provide informed consent. Participants 18 years and over provided informed consent, whereas participants under 18 years provided informed assent and caregiver consent. We randomly assigned study participants to one of two arms: a control group receiving the standard-of-care, and the intervention arm, involving 12 virtual group chat sessions covering various topics, such as treatment adherence, stigma and self-efficacy, relationships, goals and future planning, and sexual and reproductive health. For each intervention group chat, two facilitators—both bi-lingual, female South Africans trained in social work, education, and HIV care (authors TS and NN)—co-led the modules for the adolescents. The facilitators also ran a separate group chat for the caregivers of the participants which addressed the same material each week. Full information about InTSHA can be found in the intervention protocol [41].

### Study design and sample selection—SRH qualitative study

**Fig. 1:**
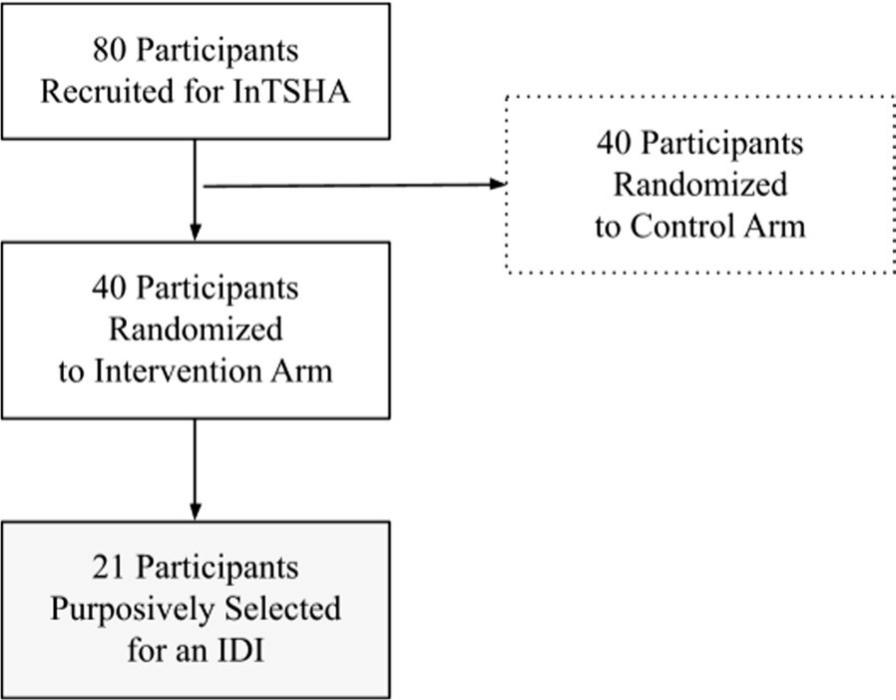
Study sample (n = 21)

From September to December 2021, we used purposive sampling via telephone to select adolescents who had participated in the InTSHA intervention and completed the modules on Sexual and Reproductive Health and Gender and Sexuality (referred to as the ‘SRH modules’) (Fig. 1). Inclusion and exclusion criteria were the same as that of the InTSHA intervention. Research assistants utilized Maximum Variation Sampling to recruit a diverse representation of age, sex, and experiences amongst adolescents who participated in the intervention [42]. No participants refused to participate, and enrollment continued until thematic saturation was met (i.e., no new themes or codes were identified). We adhered to the Consolidated Criteria for Reporting Qualitative Research guidelines [43] (Additional file 1: Appendix S1).

### Data collection

Each participant completed a 30- to 60-min in-depth interview (IDI) in a private room at KwaMashu Community Health Center. The IDI guide (Additional file 2: Appendix S2), with questions based on the intervention content, was organized to explore: perceptions of sexuality, reproductive health, and cultural norms about sex; personal experiences with sexuality, relationships, and conversations about SRH, and opinions of the intervention topics and online format. Interviewers included a multi-lingual, Bachelor’s-level, South African research assistant (author TS) and an English-speaking, Master’s-level, American research fellow (author SB), both female, who conducted IDIs in the participant’s preferred language, either English or *isi*Zulu. For each interview, one interviewer conducted and audiotaped the interview while the other took field notes. The interviewers were not associated with the clinic and were not involved in clinical care but had formed a longitudinal relationship with the participants as intervention facilitators and emphasized confidentiality, openness, and honesty throughout the study. Participants were reimbursed 250 Rand (~ $17 USD) per South African Research Counsel guidelines for transportation to and from the clinic and their time spent conducting the IDI.

### Data analysis

A bilingual research assistant who did not participate in the interviews (author MK) transcribed the audiotaped interviews in English. The primary interviewer reviewed the English transcriptions alongside the recording and interview notes for accuracy. After transcription, all participant identifiers were removed, and transcripts were stored on password-encrypted computers. Data analysis followed a thematic analysis framework [44]. Two team members (authors SB and BZ) established a final qualitative codebook through open discussion until consensus definitions were created. Team members thematically coded and analyzed the interview content into the following five parent codes: (1) Knowledge and attitudes about sexuality, (2) Knowledge and attitudes of cultural norms, (3) Romantic and sexual relationships, (4) Communication about SRH, and (5) Impressions of InTSHA. Two research team members (authors SB and MF) used thematic analysis to independently apply the coding schema to each of the 21 transcripts using Dedoose qualitative analysis software (Dedoose Version 9.0.17, Los Angeles, 2021), following a structured, hierarchical coding process, to assess perspectives of SRH before and after the intervention until thematic saturation was reached. Additional research team members (authors TS and BZ) reviewed the coded transcripts to ensure consistency between qualitative data and results before content was exported from Dedoose. The team utilized the code report to outline thematic analysis, and then author SB systematically reviewed all relevant quotes to select the data to be included in the paper.

### Ethical approvals

The Biomedical Research Ethics Committee of the University of KwaZulu-Natal, KwaZulu-Natal Department of Health, Mass General Brigham (formerly Partners HealthCare) Research Ethics Board, and Emory University Institutional Review Board approved this study.

## Results

We interviewed 21 adolescents with a mean age of 16.6 years (range: 15–19), 61.9% of whom were female (Table 1). Most (90.4%) adolescents conducted their interviews in *isi*Zulu. Thematic coding resulted in two major themes: (1) sources of SRH knowledge for APHIV outside of InTSHA, and (2) the role of InTSHA on SRH knowledge, attitudes, and behaviors.

**Table 1:** Descriptive characteristics of study sample (n = 21)

Variable	n (%) or Median (range)
**Sex**	
Male	8 (38.1%)
Female	13 (61.9%)
**Age (years)**	16.6 (15–19)
**Interview language**	
isiZulu	19 (90.4%)
English	2 (9.6%)
**Relationship experience**	
No relationship experience	10 (47.6%)
At least one relationship	11 (52.4%)
**Education level**	
Grade 8–9	7 (33.3%)
Grade 10–12	13 (61.9%)
Not in school	1 (4.8%)

### Sources of SRH knowledge for APHIV outside of InTSHA

#### Adolescents have a variety of personal relationship experiences

In the IDIs, some participants discussed having never been in a romantic or sexual relationship. Many did not wish to enter relationships this early in their lives, fearing the consequences of sex or opting to focus on school and friendships instead.

*“I won’t rush to have sex until I am 21 years old. I don’t even desire it.” (Female, 19).*

For those who already had relationship experiences, some described healthy relationships involving communication, trust, and conflict resolution. In one example, this involved HIV-status disclosure.

*“We were taking the same medication [...] so I felt like I could trust him”* (Female, 15).

While most relationships described were heterosexual, some described being in *“a same-gender relationship”* (Female, 15).

Others described unhealthy relationships. These involved cheating: *“My first girlfriend was seeing another guy”* (Male, 18), lack of commitment: *“She was always claiming to be busy”* (Male, 16), and pressure: “*It was moving too fast. I thought I was going to get pregnant”* (Female, 16).

#### Adolescents receive piecemeal sexual education that is often incorrect and stigmatizing

Adolescents described their limited sources of information about SRH in their everyday lives coming from caregivers, technology, and peers.

Many adolescents reported feeling a sense of discomfort at the idea of discussing SRH with family, expressing: “*I wouldn’t know where to start”* (Female, 19), “*It is just not in me yet”* (Male, 16), and “*I just feel nervous”* (Male, 18). In explaining why he had not spoken to his caregivers about sex, one adolescent reported: “*They will think I am already sexually active. They won’t believe that I am just curious”* (Male, 15).

Occasionally, adolescents reported desiring such conversations with caregivers, but said that “*old-fashioned and overprotective”* (Female, 16) familial norms prevent them from occurring.

*“The way I am brought up, my mother doesn’t communicate. When you make a mistake, she hits you, but she won’t have a deep conversation with you about being a female. Mom is not an open person”* (Female, 16),

If discussions about sexuality were present at home, adolescents reported that they were often derogatory or even threatening, preventing open communication about healthy relationships. Many adolescents discussed having protective family members who often reiterate the negative consequences of sex, such as early pregnancy: “*My granny tells me to behave so I don’t have a child while I’m still young”* (Female, 18) and educational disturbances: *“My mother tells me that I should just focus on my studies”* (Female, 15). Rather than suggesting abstinence or putting off relationships, adolescents said that some family members deliver messages of harm-reduction, such as “*to have one partner, not many partners”* (Male, 18) and “*to use condoms and contraception and avoid sleeping around”* (Female, 17).

Because of the limited exchange of information about SRH at home, one participant reported learning about sex through technology, including pornography and internet searches.

*“I first heard about sex when me, my sister, and my friend decided to go to a boy’s house to watch a porn DVD. I didn’t understand, but all I knew is that we also wanted to do this thing. Us girls then decided to do a role play, one to be a man and one to be a woman. We got caught and we got a hiding”* (Female, 16).

Other times, adolescents reported turning to technology to learn about sexuality when they had no one else to turn to for information.

*“I am not allowed to watch soap operas with lovers when my mother is around, but when I am alone, I watch them and learn from them. I also use the phone internet to learn about what is happening in real life”* (Female, 16).

Due to limited information at home, APHIV reported often seeking out sex education from peers. Friends, they reported, are often a positive source of information about SRH in contrast to family or the internet. Participants reported relying on their friends to decide the right time to engage in sex: “s*ome friends say we are still too young”* (Female, 19), and: “*we tell each other how much of a burden it can be to have a child while young”* (Female, 17). Other adolescents recalled receiving advice from peers on dealing with coercion in relationships: “*my friends said to just tell him how you feel and walk away”* (Female, 17), and navigating their first relationships: *“I was shy, but I opened up to my friend and she guided me”* (Female, 18). One adolescent reported being the friend that typically gives advice to others: “*I am a person who they can communicate with easily, so I advise them depending on their situations”* (Female, 16). Others described a mutual exchange of information in a group setting: “*We sit down as guys and talk about what can build or destroy relationships”* (Male, 18), and “*My friends and I talk about girls and the way they behave”* (Male, 15).

However, these unmoderated settings for adolescents discussing SRH often lead to peer pressure, gossip, and misinformation. Adolescents reported discrimination in discussions about SRH at school, saying *“my classmates will joke about sex and just make fun of it. Sometimes they also joke around about HIV”* (Female, 18). Further, many reported a lack of confidentiality, and even the spread of false gossip, amongst classmates. One participant reflected:

“*My relationship ended so badly, because she told all her friends that I was too scared to have sex. And her friends told my friends. I was so mad, so I just left her and got out of school and got away from it all”* (Male, 18).

Some reported that peer pressure made them rush into sexual activity: “*my friends put pressure on us, telling her to kiss me”* (Male, 15). When asked to discuss the mechanisms of peer pressure, the same adolescent explained:

“*We do things for the hype. In my community, once you start doing something, you feel accepted and get compliments from friends”* (Male, 15).

Male adolescents said that sex is spoken about in an aggressive way in their community: *“they use a lot of words about sex. They end up cursing each other about it”* (Male, 16). Another adolescent described the lack of confidentiality and trust with community members, explaining: “*I cannot tell things to a person from outside, because you never know if they want to gang up on you or tell secrets about you”* (Male, 18). Overall, adolescents have a desire to discuss sex, sexuality, and health, but their current sources of information are limited.

### The role of InTSHA on SRH knowledge, attitudes, and behaviors

#### Adolescents described some of the lessons they learned from the SRH modules

Many participants described gaining a holistic understanding of SRH through the mHealth intervention that changed their attitudes, knowledge, and behaviors. Table 2 shows how participants responded when asked, “What has changed for you since completing the SRH modules?”, in which adolescents describe new information, changed attitudes, and healthier behaviors in their lives after participation in the WhatsApp modules.

**Table 2:** Participant responses to the question “What has changed for you since completing the SRH modules?”

Post-Intervention Changes	Quote	Identification
Changes in attitude	*“You have to talk to your partner and have an agreement”*	Male, 15
	*“A healthy relationship has trust and communication”*	Male, 16
	*“I learned not to care about what people say, and not to rush things”*	Male, 18
Changes in knowledge	*“Sex at an early age is not good because you get pregnant while your peers continue at school”*	Female, 15
	*“We must always use protection when having sex”*	Female, 18
	*“Now I know sexuality involves two people who agree to be involve in sex, or other things you can agree to do”*	Female, 16
Changes in behavior	*“I don’t harass girls anymore due to what I learned from the group”*	Male, 15
	*“I learned how we shouldn’t force each other to have sex, and if someone says no, we should respect that”*	Female, 19
	*“It was a good thing to educate us about sex because most of us might end up doing wrong things”*	Male, 15
	*“I realized that if a person puts pressure, you need to distance yourself from them. Be brave enough to say you are not ready and for the person to respect your decision”*	Male, 15

#### InTSHA is a conversation starter

Adolescents said that the mHealth intervention allowed them to begin conversations about SRH with their caregivers, where discussion of SRH had previously been lacking. Adolescents reported that having family around when they participated in the modules could be an asset to their comprehension of the content: “*you are sometimes in the house with other people and you can chat to them as well”* (Male, 15). Referencing the simultaneous modules led with caregivers of ALHIV, one participant said: “*My auntie knows about the group, she is in the group”* (Male, 18). When asked whom they turned to when they did not understand content from the modules, one participant said, “*My mother helped me and explained”* (Male, 16). Similarly, another adolescent reported *“I asked my parents when I didn’t know”* (Male, 15).

However, others viewed the lack of privacy in the home as a barrier to participation. “*I was not comfortable talking about HIV in front of the people who live with me”* (Female, 16). Another claimed phone-sharing was a challenge to confidentiality in sensitive SRH conversations—“*we fear sharing phones with parents and parents going through the phones*” (Male, 16).

#### Adolescents left the intervention with additional questions

During the IDIs, adolescents were asked “What skills or knowledge would you like to develop more?” and “What do you still feel confused or uncomfortable about?”. Some of their responses to remaining SRH questions and unmet health needs are highlighted in Table 3.

**Table 3:** Participant responses to the questions “What skills or knowledge would you like to develop more?” and “What do you still feel confused or uncomfortable about?”

Topic	Question	Identification
HIV disclosure	“*Is it ok to tell your girlfriend that you are HIV positive?”*	Male, 15
	*“Are we able to tell our partner after being together for a long time? Are we forced to disclose?”*	Female, 18
HIV transmission	*“As people taking medication, are we able to be sexually active?”*	Female, 18
	*“If you take your medication daily, can the virus be transmitted?”*	Male, 16
	*“If a condom bursts, can the girl get the virus?”*	Female, 19
Sexual abuse	*“I want to learn more about sexual abuse”*	Male, 16
Age gap relationships	*“How can a young girl be in a relationship with someone old enough to be their father?”*	Male, 15
Reproductive choices	*“With abortion, are there any major after-effects that you go through?”*	Female, 17
	*“How long does contraception take to work?”*	Female, 17
Relationships and communication	*“We must learn how to treat each other as people”*	Female, 18
	*“You must teach us the way we should behave as teenagers”*	Male, 17
	*“We need to know about relationships, so that when we reach the right age, we can understand what life is really like”*	Female, 17
	*“Youth must learn ways to build self-confidence, see their own worth, have dignity and self-respect, be straightforward about what we want, and not be influenced by peer pressure”*	Female, 16

#### InTSHA fostered an empowering environment

Adolescents appreciated how conversations with the group facilitators play a novel and critical role in their sexual health education:

“*We need mentors and people to counsel us as we start the journey of dating, like you from the clinic. You have more knowledge than most and can give us sound advice”* (Female, 18).

One adolescent reported that some participants needed the intervention more than others—“*Some of us want the help because we have had some experiences and want more knowledge”* (Female, 17). These ideas were expressed by the following adolescent, who said “*I feel comfortable and confident, and I can always go back to the group chats”* to review content from the modules (Male, 16).

Participants explained how the modules evolved both their knowledge and ways of thinking about SRH, saying “*It gave us big lessons to push us to do the right thing”* (Female, 17). Another explained: “*I didn’t know a lot about sex and now I am more educated. I was very shy but now I am not shy anymore. It helped me with confidence* (Female, 17)”.

Peer support was a unique asset of the InTSHA intervention. Some of the reasons that adolescents enjoyed the WhatsApp format when discussing SRH topics included a having a shared HIV status with others in the group: *“I feel safe and comfortable talking about my status”* (Male, 17), being a typically introverted person in group settings: “*those that are like me will feel comfortable when no one is looking at them”* (Female, 18), and emphasizing comfort: “*everyone felt free and open”* (Female, 17).

One adolescent reported that the comfort he developed with the group during the initial in-person session continued during the mHealth intervention: “*I enjoyed that we worked as a team. I didn’t know that people were so open like that and have the guts to tell us what is going on in their lives,”* and continued throughout the virtual chat groups: “*They still talk to me in private. They ask me how life is”* (Male, 18).

Many reported enjoying the familiar technological platform to exchange sensitive information confidentially and comfortably within a group that they trust. One compared it to another popular social media platform: “*I am getting used to it, speaking to a person that I can’t see, like on Facebook”* (Female, 18). Another explained that certain adolescents may prefer the WhatsApp format because *“some people find it hard to open up in face-to-face sessions”* (Female, 18). A participant described using various features of the app outside of the group chat, such as “*private messages to others in the group* w*hen there is a word I don’t understand”* (Female, 16).

Adolescents agreed that mHealth interventions can supplement and improve their current sources of SRH information and support while initiating conversations about sexuality.

## Discussion

APHIV participants reported having a variety of relationship experiences, but having non-specific, incorrect, and stigmatizing sources of information about SRH. They usually preferred peer interactions to conversations with caregivers, which are usually perfunctory or prescriptive. Describing their experiences with InTSHA, they reported that the mHealth intervention filled gaps in their SRH education by bringing together adolescents with similar backgrounds to learn holistically about SRH. They described having engaged, bi-directional conversations that made lasting changes in their SRH attitudes, knowledge, and behaviors. While a variety of questions and remaining health needs pertaining to SRH came about during the interview (Table 3), many of these topics—such as Disclosure, Relationships and Communication, and HIV Knowledge—were the sole focus of other modules that had yet to appear during the broader intervention. Their interest in these themes shows the importance of intertwining SRH education with other forms of health education for APHIV. The ability for participants to openly ask questions about sensitive topics reveals newfound skills in SRH communication that can open up a new source of SRH knowledge in this population.

Our results bring nuance into these pre-established notions of SRH education for APHIV. Not only is traditional sexual health education in schools insufficient all over the world [45, 46], but the content and learning environment is often stigmatizing for APHIV. While classmates often spread rumors and misinformation about SRH, adolescents rely on relationships with close friends for information and support as they navigate their romantic and sexual relationships [47]. However, adolescents do not usually share their HIV status with friends, just certain family members. Although caregivers are usually dismissive about SRH with their APHIV, and especially admonitory with their daughters [48], our results show that they have the potential to be essential sources of information and resources for their children.

While existing mHealth interventions focus on the exchange of information with an expert without incorporating support from peers, InTSHA is unique in developing comprehensive, interactive sexual education specific to the unique sexual heath and support needs of APHIV. In our study, adolescents reported that mHealth can provide a supportive, less stigmatizing, mutual exchange of information and experiences about SRH. According to the adolescents, the familiar WhatsApp format fostered comfort and privacy due to the confidentiality and anonymity, bi-directional conversations that involved peer support in a moderated environment, and conversational skill-building that sparked conversations outside of the intervention. Some of these findings have been observed in other mHealth studies in different populations [49]. Participants reported that InTSHA created a group of peers with a shared background—HIV status, culture, and neighborhood—that evolved their SRH knowledge, attitudes, and behaviors. While InTSHA was not meant to be an all-inclusive curriculum of all SRH topics, adolescents reported that it bridged the gap between their knowledge and behavior by improving decision-making and communication skills. Adolescents left the modules with questions that they were able to ask other group members, study staff, and family members, sparking conversations about SRH outside of the intervention.

It is critical to contextualize these results in current events and the sociocultural environment. During the intervention, KwaZulu-Natal was facing the devastating effects of political unrest and new waves of the COVID-19 pandemic, which had drastic effects on HIV-care and in-person education around the world [50]. However, mHealth has been shown to continue to provide support and information related to chronic disease care when traditional service delivery mechanisms are disrupted [51]. Within InTSHA, participation was high in the WhatsApp groups as adolescents were able to participate from their own homes during a time when safe transportation to the clinic would have been burdensome or unsafe. Our data shows that InTSHA allowed APHIV to feel safe, open, and confident as they learned together.

## Limitations

This study has several limitations. This qualitative study consisted of 21 ALHIV who completed the mHealth intervention *InTSHA*. Due to a short follow-up period and a lack of control group, we cannot report on long-term knowledge or behavior change. Future quantitative analyses will assess the impact of this intervention on a larger and more longitudinal scale, with comparisons drawn between the intervention and control groups at various time points. Further, all participants had some access to a smartphone and mobile data, without which they could not have participated in the intervention. In other populations within this same region, a mobile health intervention may not be as feasible and acceptable due to technology and data limitations. However, smartphone coverage in South Africa is estimated to be over 90%, and nearly all of the population has data coverage, pointing to the feasibility of potential scale up [23]. Notably, adolescents in our study were all engaged in regular HIV care in their community, and thus had a level of health-seeking behavior that is not the case for many South African APHIV, which may have led to more favorable reactions to the SRH intervention. This study was meant to highlight SRH in APHIV who are engaged in care. This study presented the risk of social desirability bias, through some intrinsic factors such as providing a reimbursement, not being able to gender match the interviewers, and having interviewers who had been involved in the intervention facilitation and creation. However, we took steps to mitigate such bias, such as beginning the interview with an emphasis on seeking honest feedback, forming longitudinal relationships with participants to build trust and honesty, and emphasizing confidentiality throughout the intervention and interview. Relatedly, topics discussed were often sensitive, and some adolescents appeared less open sharing details of their relationships and sexual behaviors with interviewers. This could have limited possible alternative perspectives. To combat this, all efforts were made in the interview questions, format, and confidentiality to make participants feel comfortable sharing their experiences.

## Conclusion

Many South African APHIV receive inadequate, fragmented sexual education from a combination of peers, caregivers, and teachers that is not targeted to those living with HIV. The use of mHealth interventions for holistic sexuality education, specifically tailored to the unique SRH needs of APHIV, can normalize sexuality, expand access to correct SRH information, and begin conversations to improve the sexual health knowledge and behaviors of APHIV.

## Supplementary Information

**Additional file 1.** Consolidated criteria for reporting qualitative research (COREQ) checklist.

**Additional file 2.** Sexual and reproductive health interview guide.

## Data Availability

Data and qualitative node summaries are available upon request.
