# Supplementary material for: “I am not shy anymore”: A qualitative study of the role of an interactive mHealth intervention on sexual health knowledge, attitudes, and behaviors of South African adolescents with perinatal HIV
Source: Reprod Health. 2022 Dec 1;19:217. doi: 10.1186/s12978-022-01519-2 (PMC9713189; doi:10.1186/s12978-022-01519-2)
Supplement: Supplementary file 2 — Additional file 2. Sexual and reproductive health interview guide. [file 12978_2022_1519_MOESM2_ESM.pdf]

Appendix 2: SRH Interview Guide

Hi! Thank you so much for joining me today. My name is [NAME] and I am a researcher at [INSTITUTION]. I am excited to speak with you about some of the topics you learned about in the Sexuality and Reproductive Health Module of InTSHA. I would love to hear as many of your thoughts as you feel comfortable sharing today, but if you ever feel uncomfortable, you can skip questions or we can stop the interview altogether. Anything you say here will be kept confidential, which means your name will never be attached to your answers. I expect this conversation to take around one hour. Do you have any questions before we begin?

First we will talk about sexuality and cultural norms:

- What does the word sexuality mean to you? (answer: sexuality refers to sexual activity, or a person's identity in terms of what gender they are attracted to)
    - How did you first learn about sex? How old were you? Who taught you? (school, friends, family, doctor, nurse, counselor)
    - Who do you feel most comfortable speaking with about your sexual health, like accessing contraception, relationship advice? (teacher, friends, family, doctor, nurse, counselor, internet)? Why?
- What does the phrase "cultural norm" mean to you (answer: a societal rule, value, or standard that describes accepted and appropriate behavior within a culture)
    - What are some cultural norms about sexuality in your family?
    - What are some norms about sexuality in your school or community?
    - How are norms about sexuality different for boys and girls?
    - Who enforces norms about sexuality?
    - Where do you think the norms come from?

Next, we will talk about romantic and sexual relationships.

- What are some signs of a healthy relationship?
- What are some resources that teens need before they are in a relationship?
- What are some signs of an unhealthy relationship?
- How would you respond if someone is pressuring you to do a physical act that you don't want to do?
- Do you now, or have you ever, had a boyfriend or girlfriend?
    - **IF YES:** Tell me about this relationship.
    - How did you communicate about physical acts such as kissing, touching, or sex?
    - How did you talk about your relationship to your friends?
- How did you talk about your relationship to your family members?
    - **IF NO:** When do most of your peers begin having relationships?
    - When would you be ready to be in a relationship?

Finally, we will discuss the InTSHA intervention that you participated in.

- What were your thoughts on the sexual and reproductive health module in InTSHA?
    - How did you feel about the WhatsApp format?
    - What difficulties did you face when accessing or participating in the online module?
    - How did the SRH online module compare to any in person sexual health education you have received (for example, Life Orientation)?

- What has changed since completing the SRH module? (knowledge, behaviors, or attitudes)
- What skills or knowledge would you like to develop more? (consent, communication, and accessing health resources)
- What do you still feel confused or uncomfortable about?
- What would you like more information about or to discuss further?

Thank you so much for this helpful conversation. We will use your feedback to improve the InTSHA intervention, as well as sexual health education for many more adolescents in the future. Have a great day!
